# Evolutionary selection of pestivirus variants with altered or no microRNA dependency

**DOI:** 10.1093/nar/gkaa300

**Published:** 2020-05-06

**Authors:** Konstantinos G Kokkonos, Nicolas Fossat, Louise Nielsen, Christina Holm, Wytske M Hepkema, Jens Bukh, Troels K H Scheel

**Affiliations:** 1 Copenhagen Hepatitis C Program (CO-HEP), Department of Infectious Diseases, Hvidovre Hospital, Hvidovre 2650, Denmark; 2 Department of Immunology and Microbiology, Faculty of Health and Medical Sciences, University of Copenhagen, Copenhagen 2200, Denmark; 3 Laboratory of Virology and Infectious Disease, The Rockefeller University, New York, NY 10065, USA

## Abstract

Host microRNA (miRNA) dependency is a hallmark of the human pathogen hepatitis C virus (HCV) and was also described for the related pestiviruses, which are important livestock pathogens. The liver-specific miR-122 binds within the HCV 5′ untranslated region (UTR), whereas the broadly expressed let-7 and miR-17 families bind two sites (S1 and S2, respectively) in the pestiviral 3′ UTR. Here, we dissected the mechanism of miRNA dependency of the pestivirus bovine viral diarrhea virus (BVDV). Argonaute 2 (AGO2) and miR-17 binding were essential for viral replication, whereas let-7 binding was mainly required for full translational efficiency. Furthermore, using seed site randomized genomes and evolutionary selection experiments, we found that tropism could be redirected to different miRNAs. AGO cross-linking and immunoprecipitation (CLIP) experiments and miRNA antagonism demonstrated that these alternative variants bound and depended on the corresponding miRNAs. Interestingly, we also identified miRNA-independent variants that were obtained through acquisition of compensatory mutations near the genomic 3′ terminus. Rescue experiments demonstrated that miRNA binding and 3′ mutagenesis contribute to replication through mutually exclusive mechanisms. Altogether, our findings suggest that pestiviruses, although capable of miRNA-independent replication, took advantage of miRNAs as essential host factors, suggesting a favorable path during evolutionary adaptation.

## INTRODUCTION

Bovine viral diarrhea virus (BVDV), a positive-strand RNA virus in genus *Pestivirus* of the *Flaviviridae* family, is a major pathogen of cattle causing vast economic losses to the livestock industry (1). Classical swine fever virus (CSFV) and border disease virus are other important pathogens for pigs and sheep, respectively, in the *Pestivirus* genus (2). The BVDV genome of ∼12 300 nucleotides (nt) contains short 5′ and 3′ untranslated regions (UTRs) and a large single open reading frame (ORF) encoding a polyprotein that is post-transcriptionally processed into distinct structural and non-structural proteins (1). Unlike eukaryotic mRNAs, the BVDV 5′ UTR lacks a cap structure and instead harbors an internal ribosomal entry site (IRES) recruiting ribosomes for translation. The 3′ UTR lacks a poly-A tail and instead contains conserved terminal stem loops (SLI–III) (3). BVDV exists as two biotypes: non-cytopathic (ncp) and cytopathic (cp). Transient infection of naïve animals with either biotype causes only mild symptoms, and after seroconversion, the animals become immune. On the other hand, infection of the embryo with an ncp strain can lead to immunotolerance and persistence (4). The emergence of cp variants from ncp variants in persistently infected animals is consistently associated with a lethal outcome, referred to as mucosal disease. These cp variants can emerge from ncp variants by deletion or duplication of viral sequences, by acquisition of mutations or by host sequence insertions through RNA recombination (5).

MicroRNAs (miRNAs) are short (∼22 nt) non-coding RNAs that fine-tune cellular gene expression post-transcriptionally. Argonaute (AGO) 1–4 and other proteins, which together constitute the RNA-induced silencing complex (RISC), are loaded with miRNAs and bind cellular mRNAs, typically to the 3′ UTR. Base pairing between the miRNA (nt 2–7) and its 6mer seed site leads to destabilization and/or translational repression of the target mRNA (6). Additional pairing of the neighboring nucleotides strengthens this effect.

In contrast, certain RNA viruses use miRNAs to promote and direct viral infection. Viral dependence on host miRNAs was first shown for hepatitis C virus (HCV) (7–9). HCV is one of the most important human pathogens with >70 million people persistently infected and at increased risk of developing chronic liver diseases, including cirrhosis and cancer (10). HCV recruits the liver-specific miR-122 to two seed sites located in its 5′ UTR, which enhances RNA stability, translation and replication (11,12). All clinical HCV isolates, as well as equine (EqHV/NPHV), bovine (BoHV) and rat hepaciviruses (RHV), are stimulated by miR-122 (13–16). During the last 30 years, a major effort has been made to develop HCV antiviral therapies (17,18), including antagonists targeting the miR-122 interaction (19–21). Although miR-122 inhibitors in HCV therapy might be outcompeted by successfully implemented directly acting antivirals, they have demonstrated the huge potential of miRNA-based therapeutics.

Recently, we surprisingly found that BVDV and CSFV also depend on cellular miRNAs. However, in these cases, miRNAs of the let-7 and miR-17 families (miR-17, miR-20, miR-93 and miR-106) bind the viral 3′ UTR (22). The seed sites for let-7 and miR-17 are perfectly conserved for all sequenced pestiviruses, except for the recently identified atypical porcine pestiviruses. Although miRNA binding occurs at a different genomic region than for HCV, it also stimulates viral RNA accumulation; miRNA binding upregulates translation and protects the genome from degradation, while miRNA antagonism or seed site mutations led to substantial reduction or complete abrogation of replication (22). Replication of such mutants could be rescued by trans-complementation with the corresponding miRNA mimics (22). While HCV is restricted to infect the liver, partially due to its dependence on miR-122, pestiviruses have a broad tissue tropism correlating with broad expression of let-7 and miR-17 (23). Experimental development of miRNA-independent HCV variants has been reported (13,24,25), whereas the requirements and implications of miRNA binding for pestiviruses have not been investigated further. Therefore, studying the relationship between miRNAs and pestiviruses constitutes a unique opportunity to gain better understanding of virus–miRNA interactions and to advance RNA therapeutics for host RNA-dependent viruses (8).

Here, we aimed to elucidate the interaction between BVDV and host miRNAs. We delineated the importance of each of the two miRNA seed sites for translation and replication. To understand the plasticity of the BVDV genome, we mutated and randomized the miRNA seed sites. BVDV could under specific experimental conditions adapt to different miRNAs or even become miRNA independent. The resulting variants are the first among the typical pestiviruses to not bind miR-17, demonstrating that pestiviruses could have escaped miRNA dependency during adaptation, but still evolved to depend on miR-17.

## MATERIALS AND METHODS

### Cell culture

Madin–Darby bovine kidney (MDBK) cells were maintained in Dulbecco’s modified Eagle medium (DMEM, Invitrogen) supplemented with 0.1 mM non-essential amino acids, 0.1 mM sodium pyruvate and 10% horse serum (HS).

### Construction of virus and reporter plasmids

Mutants of the BVDV NADL strain were engineered using QuikChange Lightning Multi Site-Directed Mutagenesis Kit (Agilent Technologies), In-Fusion HD Cloning Kit (Clontech, Takarabio) and standard polymerase chain reaction (PCR) and cloning methods. The randomized genomes were created using AccuPrime Taq DNA Polymerase high fidelity (Thermo Fisher Scientific) and primers with degenerate nucleotides in a long PCR to generate an amplicon starting with the T7 promoter followed by the complete BVDV genome with cycling parameters of 94°C for 2 min followed by 35 cycles of 94°C for 15 s, 58°C for 30 s and 68°C for 12 min. The templates used for the different randomized variants are shown in Supplementary Table S1, and primer sequences in Supplementary Table S2. Transformation of BVDV plasmids was done into Sure 2 supercompetent cells (Agilent Technologies) and small- and large-scale production prepared using QIAprep Spin Plasmid Miniprep and QIAfilter Maxiprep (Qiagen) after growth in terrific broth medium, as was described previously (22). The bidirectional reporter was constructed by restriction digest cloning. Specifically, monocistronic BVDV-Fluc (22) was digested with NaeI followed by mung bean nuclease (NEB) treatment to create blunt ends. In a second digestion, the resulting fragment was digested with KpnI to generate the vector fragment. The monocistronic NPHV-Rluc reporter (26) was digested with XbaI and treated with mung bean nuclease and sequentially digested with KpnI to generate the insert. The vector and insert were ligated to create the bidirectional reporter. The resulting renilla luciferase (Rluc) flanked by the NPHV UTRs was used as an internal control, while firefly luciferase (Fluc) flanked by the BVDV UTRs was used to measure translation. After linearization with SbfI, treatment with mung bean nuclease generated the exact BVDV 3′ end and T7-mediated transcription simultaneously produced both reporter RNAs, which were then transfected into MDBK cells.

### RNA transcription, electroporation and transfection

To produce viral RNA for electroporation of BVDV, DNA was linearized with SbfI and purified on DNA Clean & Concentrator—5 columns (Zymo Research). RNA was produced using T7 RiboMAX™ Express Large Scale RNA Production System (Promega). The RNA was DNase treated for 30 min on ice, purified using RNeasy mini kit (Qiagen) and quantified and visualized using Qubit RNA HS Assay (Invitrogen) and gel electrophoresis. A Gene Pulser Xcell Electroporation System (Bio-Rad) was used for RNA electroporations. Typically, 5 μg RNA was added to 400 μl suspension of 8 × 10^6^ MDBK cells followed by two pulses of 100 μs, 900 V over 5 s. Trans-complementation with mutant miRNA mimics (GE Dharmacon, Horizon) or tiny LNAs (Exiqon, Qiagen) was done 24 or 48 h pre-electroporation, during the electroporation procedure by adding the oligos directly in the electroporation mix and every other day thereafter by transfection. Briefly, for reverse transfection, Lipofectamine RNAi/MAX (Thermo Fisher Scientific) was mixed with small RNAs or LNAs in Opti-MEM (Thermo Fisher Scientific) and the mix was incubated for 10 min at room temperature. The solution was mixed with media containing MDBK cells and seeded in the appropriate plate/dish. For transfection of seeded cells, the same procedure was followed but plain cell-free medium was instead added to the RNAi/MAX-LNA/mimic solution. For siRNA treatment, cells were reverse transfected into flasks with RNAi/MAX (siAGO1_1_ at 2 nM against AGO1, siAGO2_1_ and siAGO2_2_ at 10 nM each against AGO2 or siControl at 10 nM). After 48 h, cells were reverse transfected with the same siRNA a second time and plated into 24-well plates. Sequences for LNA, mimics and siRNAs are given in Supplementary Table S3.

### Protein extraction and western blotting

After 72 h of treatment with siRNAs, cells were infected with virus. A replica plate was used to purify protein from cell pellets. Briefly, cells were trypsinized, resuspended in cold phosphate-buffered saline and the pellets were frozen at −80°C. To purify cellular protein, cells were lysed with RIPA buffer (Thermo Fisher Scientific), supplemented with cOmplete protease inhibitor cocktail (Merck), followed by RQ1 DNase (Promega) treatment. Protein concentration was measured with Pierce BCA protein assay kit (Thermo Fisher Scientific). AGO1 was detected with polyclonal Anti-Argonaute-1 Antibody (Thermo Fisher 711755) at 0.5 mg/ml and AGO2 with Anti-Argonaute-2 Antibody (Abcam ab32381) at 1 mg/ml. Actin was used as a control and was detected with monoclonal Anti-Actin Antibody (Santa Cruz sc-47778) at 200 μg/ml.

### Sequencing of viral RNA from supernatant

Viral RNA from supernatants was extracted using TRIzol LS reagent (Thermo Fisher Scientific) and purified with RNA Clean & Concentrator—5 columns (Zymo Research). For sequencing of the BVDV 3′ UTR, cDNA was synthesized using SuperScript III (Life Technologies) and a BVDV specific primer (TS-O-00058) in a gradient from 50 to 55°C for 60 min, followed by treatment with RNase H and T1 (Thermo Fisher Scientific) for 20 min at 37°C. PCR was performed using AccuPrime Pfx supermix (Thermo Fisher Scientific) with cycling parameters of 95°C for 2 min followed by 35 cycles of 95°C for 20 s, 56°C for 30 s and 68°C for 1 min and primers TS-O-00036 and TS-O-00058. For ORF sequencing, cDNA was synthesized using Maxima H Minus Reverse Transcriptase (Thermo Fisher Scientific) at 50°C for 2 h followed by treatment with RNase H for 20 min at 37°C. The same PCR as described earlier was used, but instead with 12 min of extension and primersTS-O-00787 and TS-O-00788. The amplified viral DNA was purified with Zymoclean Gel DNA Recovery (Zymo Research) and sequenced (Macrogen). Analysis of individual clones was done using the Zero Blunt TOPO PCR cloning kit (Thermo Fisher Scientific). Primer sequences for these procedures are listed in Supplementary Table S2.

### Total RNA extraction and titration

Intracellular RNA was extracted with RNeasy mini kit (Qiagen) using an on-column RNase-free DNase set (Qiagen). Quantification of viral RNA was done by running a qPCR with TS-O-00061, TS-O-00062 and TS-O-00063 recognizing BVDV and TS-O-00094, TS-O-00095 and TS-O-00096 recognizing ribosomal protein S11 (RPS11) mRNA, which was used as an internal control, on a LightCycler 96 (Roche) using TaqMan Fast Virus 1-Step Master Mix (Thermo Fisher Scientific) in a one-step RT-qPCR at 50°C for 30 min, 95°C for 5 min followed by 40 cycles of 95°C for 15 s, 56°C for 30 s and 60°C for 45 s. Standard curves for BVDV and RPS11 were generated from *in vitro* transcribed RNA from plasmids, quantified (Qubit) and diluted to cover the range of 10^8^–10^1^ genome equivalents (GE)/μl. Primers and probes are given in Supplementary Table S2.

### Viral infectivity titration and monitoring of infection

For plaque-forming unit (PFU) assays, cells were plated in six-well plates and 1 day later infected with 10-fold dilution series for 1 h rocking before overlay with Avicel (2.4%) mixed with 2× complete DMEM solution and HS to 10%. Plates were incubated at 37 C for 48 h until fixation with 3.7% formaldehyde and were stained using crystal violet. Alternatively, BVDV was quantified using end-point dilution: 10^3^ MDBK cells were plated per well of a 96-well plate 1 day before inoculation with 10-fold dilution series of BVDV. After incubation for 96 h, the wells with cytopathic effects (CPEs) were scored as positive. Titers were calculated according to tissue culture infectious dose-50 (TCID_50_) method.

### Deep sequencing

To create libraries for deep sequencing of the seed sites of randomized viral genomes, cDNA was produced as described above from intracellular RNA for early time points (6–24 h) and extracellular RNA for later time points (48–120 h). The cDNA was amplified using AccuPrime Pfx supermix (Thermo Fisher Scientific) with cycling parameters of 95°C for 2 min followed by 30 cycles of 95°C for 20 s, 55°C for 30 s and 68°C for 1 min. Custom Illumina compatible BVDV adaptor primers were used for the PCR. A nested PCR was done with the same parameters but for 20 (late time points) or 40 (early time points) cycles using Illumina specific adapter oligos (Supplementary Table S2). The amplified DNA was purified with Zymoclean Gel DNA Recovery (Zymo Research), quantified (Qubit) and further analyzed on a Bioanalyzer DNA chip (Agilent). The prepared libraries were sequenced on an MiSeq using the MiSeq reagent kit v3 (150 cycles, Illumina). Bioinformatics analysis was done in Galaxy and R studio.

### Determination of genomic ends

Determination of the 5′ end of the BVDV genome was done using SMARTer RACE 5′/3′ Kit (Clontech, Takarabio). Briefly, RNA was purified from 250 μl viral supernatant as described above and cDNA was produced according to manufacturer’s instructions using a BVDV specific primer (TS-O-00039). The cDNA was amplified using 10×UPM and TS-O-01059 with a touchdown PCR with cycling parameters of 5 cycles of 94°C for 30 s, 64°C for 30 s and 72°C for 2 min, followed by 5 cycles of 94°C for 30 s, 62°C for 30 s and 72°C for 2 min, followed by 30 cycles of 94°C for 30 s, 60°C for 30 s and 72°C for 2 min. For 3′ end determination, BVDV RNA was tailed with ATP (10 mM) using Yeast Poly(A) Polymerase (Affymetrix, Thermo Fisher Scientific) at 37°C for 10 min and cDNA was made using SuperScript III (Life Technologies) and a custom-made primer with a poly-T tail (TS-O-00178) in a gradient from 48 up to 55°C, during 1 h. The cDNA was amplified using AUAP and TS-O-00913 and the product was purified with Zymoclean Gel DNA Recovery. Primers for the described procedures are listed in Supplementary Table S2.

### AGO-CLIP assay

Standard AGO cross-linking and immunoprecipitation (CLIP) was done as described (22,27). CLEAR-CLIP was used to identify the specific miRNA binding and is based on standard AGO-CLIP with modifications to enrich for miRNA target chimeras (22,28). Bioinformatic analysis was done using Galaxy (usegalaxy.org), R and Perl scripts as described previously (22).

### Luciferase reporter assays

MDBK cells were transfected with 100 ng/well reporter RNA using Lipofectamine 2000 (Life Technologies). After 4 h, supernatant was removed, the cells were lysed and the relative luciferase expression was measured using Dual-Luciferase Reporter Assay System (Promega) on a FLUOstar Omega (BMG Labtech).

## RESULTS

### Interaction with members of the miR-17, but not let-7 family, is critical for BVDV

**Figure 1.**
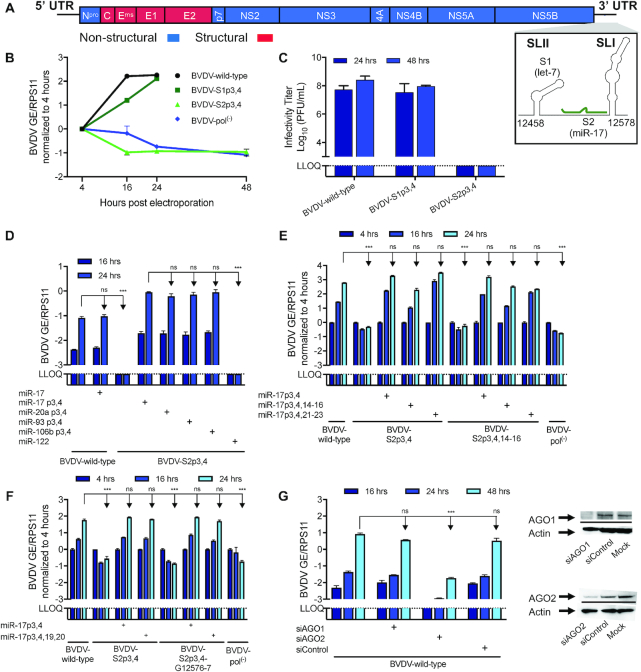
Importance of the BVDV 3′ UTR miRNA seed sites. (**A**) Schematic representation of the BVDV genome drawn to scale. The single ORF is flanked by 5′ and 3′ UTRs. The secondary structure of the NADL strain genomic 3′ end was predicted using Mfold and illustrated using VARNA. SLI and SLII are depicted and the let-7 seed (S1) and the miR-17 seed (S2) sites are indicated. Only S2 miR-17 binding was assumed for this structure. (**B**) Viral replication of BVDV wild-type and its seed site mutants (S1p3,4 and S2p3,4) after electroporation of MDBK cells measured in GE by RT-qPCR on intracellular RNA normalized to housekeeping gene RPS11 and the 4 h time point. Pol^(−)^, non-replicating control. (**C**) Virus production after infection of MDBK cells with BVDV wild-type and seed site mutants was quantified by the PFU assay. (**D**) Viral replication of BVDV wild-type or BVDV-S2p3,4 by RT-qPCR on intracellular RNA normalized to RPS11 after infection at MOI = 0.005. Trans-complementation with 10 nM miRNA mimics is indicated. (**E, F**) Viral replication of BVDV wild-type, BVDV-S2p3,4, BVDV-S2p3,4,14–16 and BVDV-S2p3,4,G12576–7 after electroporation of MDBK cells measured by RT-qPCR on intracellular RNA normalized to RPS11 and the 4 h time point. Trans-complementation with 10 nM miRNA mimic is indicated. (**G**) Left: Viral replication of BVDV wild-type after infection (MOI = 0.01) of AGO-depleted MDBK cells measured by RT-qPCR on intracellular RNA normalized to RPS11 and the 4 h time point. Right: Western blots for AGO1 and AGO2 on siRNA-treated MDBK cells. LLOQ: lower limit of quantification. Mean values (±SD), *n* = 2. Statistical differences by ANOVA to BVDV wild-type (**D**–**G**) or BVDV-S2p3,4 (**D**) are indicated for the last time point. Corrections were done according to Dunnett’s test; ns, not significant; *P ≤ 0.05; **P ≤ 0.01; ***P ≤ 0.001.

We previously reported binding of let-7 and miR-17 to the two corresponding seed sites (S1 and S2) in the BVDV 3′ UTR (Figure 1A) (22). To determine the individual importance of let-7 and miR-17 for viral viability, we electroporated and infected MDBK cells with genomic BVDV RNA (cp NADL strain) containing mutations at positions 3 and 4 of each seed site, which are known to be essential for miRNA binding (29). As previously observed, the BVDV-S2p3,4 mutant showed no replication or virus production (22). The BVDV-S1p3,4 mutant had delayed replication at early time points, but caused CPEs 2 days post-electroporation (dpe) and virus production was only minimally affected (Figure 1B and C). Combined, these experiments suggested less dependence on let-7 compared to miR-17 for BVDV viability.

To further explore the mechanism of miRNA binding to S2, we performed trans-complementation experiments using p3,4 mutants of each miR-17 family member during BVDV-S2p3,4 infection. Despite their 3′ end variation, all family members supported BVDV replication to similar levels (Figure 1D and Supplementary Figure S1A). Furthermore, this experiment showed that addition of exogenous miR-17 did not lead to further enhancement of BVDV wild-type replication (Figure 1D), demonstrating that the amount of endogenous miR-17 is sufficient for optimal replication of BVDV.

### miRNA auxiliary pairing is not required for BVDV replication

Accessory base pairing outside the miRNA seed region can exacerbate functional effects, including for the HCV/miR-122 interaction (30,31). To study the importance of auxiliary pairing for BVDV S2 (Supplementary Figure S1B), we mutagenized nucleotides 12505–7, which could pair with positions 14–16 of miR-17. To avoid interference with endogenous miR-17, these experiments were done in the BVDV-S2p3,4 context. Trans-complementation with miR-17p3,4 led to efficient viral replication even though auxiliary pairing was disrupted and miR-17p3,4,14–16 did not improve viral replication (Figure 1E). To test putative alternative auxiliary pairing between miRNA positions 21–23 and BVDV positions 12494–6 (Supplementary Figure S1B), we further trans-complemented with miR-17p3,4,21–23 mimic, again without evidence for importance of such interaction. Therefore, we concluded that auxiliary pairing plays a minor role for the BVDV/miR-17 interaction.

Having ruled out a critical role for the canonical auxiliary pairing region of the miRNA, we next investigated a potential contribution of the conserved ‘GG’ dinucleotide at nt 19–20. Since miR-122 auxiliary pairing was shown to protect the HCV 5′ end (31), we hypothesized that this miR-17 ‘GG’ motif could protect the BVDV 3′ end by interacting with its terminal Cs. We thus mutated the Cs at positions 12576–12577 and trans-complemented with miR-17p3,4,19,20 in the S2p3,4 background. These mutations did not lead to any effect on BVDV replication (Figure 1F). In contrast to HCV, BVDV therefore appears to require pairing only with the miRNA seed.

### BVDV replication depends on AGO2

For HCV, miR-122 binding is mediated through interaction with AGO2 and other RISC-associated proteins (11,32,33). To elucidate which AGO protein is important for BVDV, we used RNAi in MDBK cells to deplete bovine AGO1 or AGO2, which generally are the most important AGO proteins. Replication of BVDV wild-type was significantly affected under AGO2 but not AGO1 depletion (Figure 1G). Therefore, similarly to HCV, AGO2 is also a prerequisite for BVDV replication.

### BVDV miRNA tropism can be redirected

**Figure 2.**
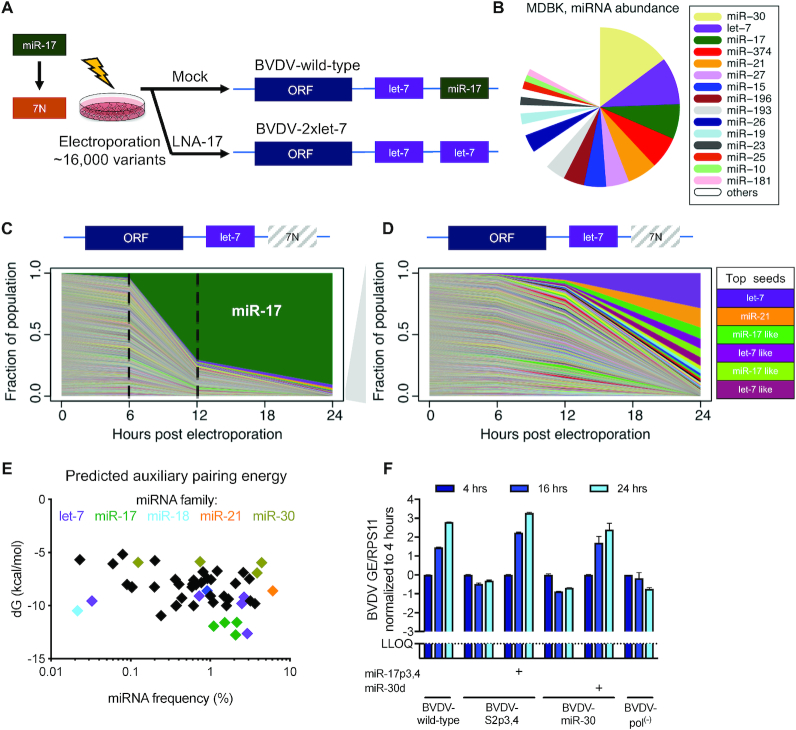
Evolutionary selection of alternative miRNA seed sites by randomization of S2. (**A**) Evolutionary selection strategy and outcome for BVDV S2 randomization experiment. Mock- or LNA-17-treated MDBK cells were electroporated with a pool of S2 randomized genomes. Shown are the genomes selected 24 h after passage of supernatant to naïve cells. (**B**) AGO-CLIP-derived miRNA abundance profile of MDBK cells. Data from (22). (**C**) Population structure over time after electroporation of BVDV S2 randomized pool in mock cells. RNA from the input pool and RNA isolated after 6 and 12 h (intracellular) and 24 h (extracellular) were deep sequenced and analyzed for miRNA seed site abundance. The fraction of each selected sequence is shown based on the S2 6mer. (**D**) Population structure of subdominant seed sites. Shown are data from (**C**) after removing genomes containing the miR-17 site. The top selected seeds are color coded according to (**B**). (**E**) Prediction of BVDV S2 auxiliary pairing energies of the top 50 miRNAs expressed in MDBK cells relative to their abundance levels. Binding energies were predicted using IntaRNA with a fixed seed region and unpaired nt 10–11, such that only auxiliary pairing was considered. (**F**) Viral replication of BVDV-miR-30 after electroporation of MDBK cells measured by RT-qPCR on intracellular RNA normalized to RPS11 and the 4 h time point. BVDV wild-type and BVDV-S2p3,4 are shown for comparison. Trans-complemented miRNA mimics at 10 nM are indicated. Mean values (±SD), *n* = 2.

It was previously shown that HCV miR-122 tropism can be redirected to miR-15 (34). To comprehensively sample miRNAs supporting BVDV replication, we randomized the S2 binding site (miRNA positions 2–8) to create a pool of 16 384 (i.e. 4^7^) variants theoretically containing all possible seed sites. Deep sequencing of the pool confirmed the presence of all 6mer and almost all 7merM8 [binding miRNA nt 2–8 (29)] seed sites (Supplementary Data S1). In supernatant collected at 2 and 3 dpe, the wild-type virus was selected (Figure 2A), confirming that an miR-17 seed site at S2 leads to the most fit virus. To force selection of alternative miRNA tropic viruses, we treated the cells with miR-17 antagonist, tiny LNA-17, before and after electroporation. Tiny LNA (LNA hereafter) targets nt 2–9 of the miRNA, therefore targeting the entire miRNA seed site family. Surprisingly, the variant selected in this experiment contained a let-7 seed at S2, thus having two let-7 seed sites (BVDV-2×let-7) (Figure 2A). To identify other putative functional sites and determine whether there is a correlation with miRNA abundance in the cell (Figure 2B), we deep sequenced early time points of intracellular viral RNA from mock-treated cells. At 12 h post-electroporation (hpe), BVDV wild-type had already outcompeted most other variants (Figure 2C). Nevertheless, consistent with the LNA-17 experiment, let-7 was the second most selected site, while other subdominant sites included miR-21, as well as mismatched or offset miR-17 and let-7 sites (Figure 2D). Surprisingly, other abundant miRNA families, such as miR-30, were not among the selected seeds. Although miR-17 auxiliary pairing showed only marginal importance (Figure 1D), stronger auxiliary pairing for the miR-17 and let-7 families compared to miR-30 was apparent when predicting auxiliary binding energies for the 50 most abundant miRNAs (Figure 2E). Electroporation of a BVDV-S2-miR-30 variant did not lead to replication or CPEs. However, supplementation with a synthetic miR-30d mimic allowed for replication levels comparable to BVDV wild-type (Figure 2F). This suggested either that endogenous miR-30 remains inaccessible to the viral RNA or that miR-30 abundance was overestimated in our assay. Thus, our results suggest that BVDV S2 miRNA tropism can be redirected to other miRNAs based on their abundance and availability.

### Context determines seed site selection

**Figure 3.**
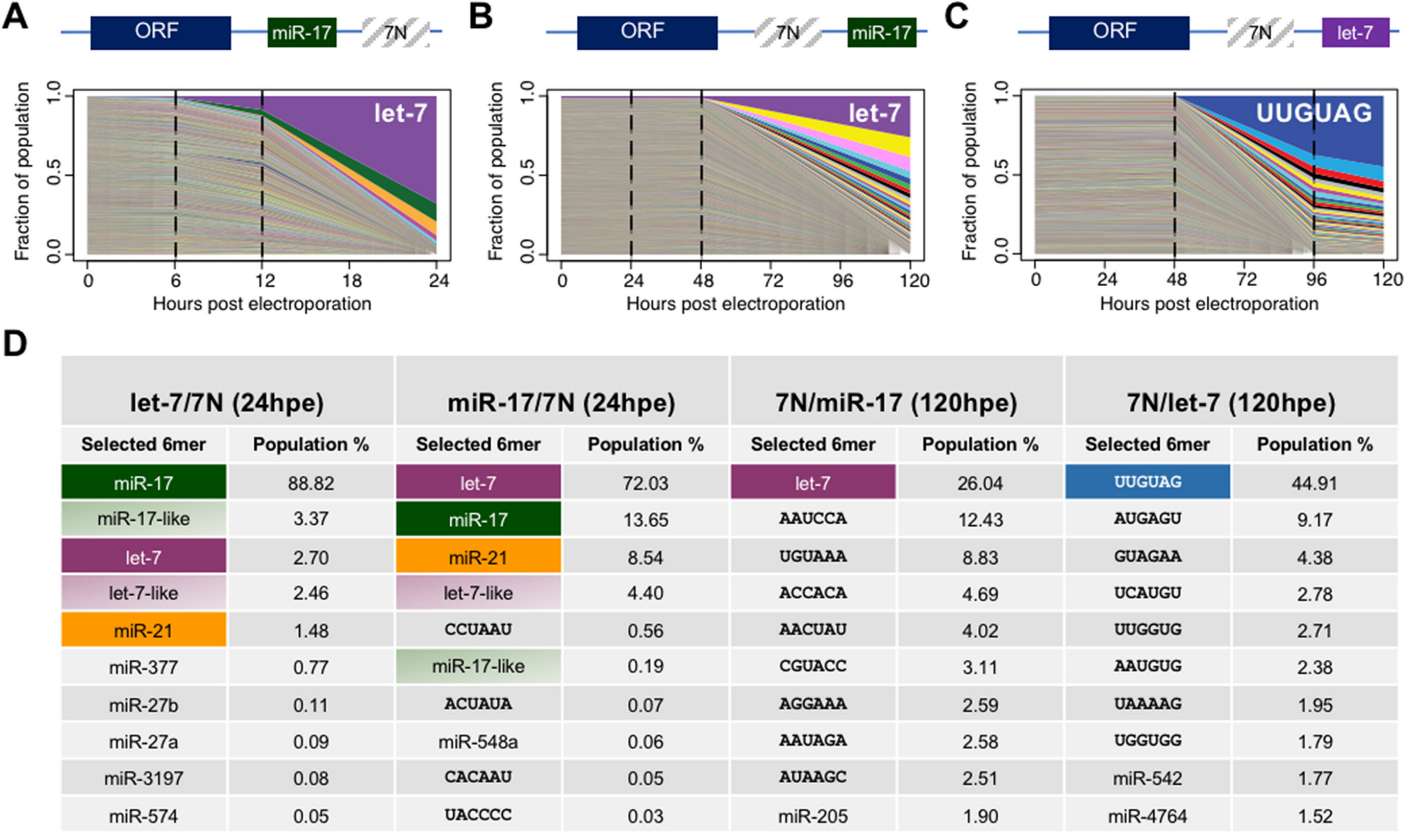
Evolutionary selection of alternative S1 and S2 sequences by randomization. Population structure over time after electroporation of MDBK cells with an S2 randomized pool in context of an miR-17 S1 site (**A**) or S1 randomized pools in context of an S2 miR-17 site (**B**) or S2 let-7 site (**C**). RNA from the input pool and RNA isolated at time points indicated by dotted lines were deep sequenced and analyzed for miRNA seed site abundance. Intracellular RNA was used for 6 and 12 h time points and extracellular RNA was used for 24 h and later time points. The fraction of each selected sequence is shown based on the seed site 6mer. Schematics of BVDV genomes are depicted above each graph. (**D**) Summary of the top 10 selected 6mer seed sites and their abundance for BVDV-let-7/7N, BVDV-miR-17/7N, BVDV-7N/miR-17 and BVDV-7N/let-7 experiments at 24 h (S2 randomizations) or 120 h (S1 randomizations) after electroporation in MDBK cells. Frequencies of sequences with only 1 nt difference from miR-17 or let-7 sites at any core 6mer position were summed and reported as miR-17- or let-7-like. The 6mer nucleotide sequence is shown whenever no corresponding miRNA seed site was identified.

We next investigated whether the two seed sites, S1 and S2, are interdependent by randomizing S2 in the context of an S1 miR-17 site. Deep sequencing 24 hpe showed that genomes harboring a let-7 site at S2 dominated the culture followed by miR-17 and miR-21 sites (Figure 3A and D). After two passages to naïve cells, however, most BVDV genomes carried an S2 miR-17 site, thereby yielding a BVDV-2×miR-17 virus. Thus, BVDV with an miR-17 seed at S2 eventually outcompeted the let-7 seed.

We next asked whether other seed sites could be selected at S1 and randomized this seed site in the wild-type context (Figure 3B). After 120 hpe, the let-7 site (wild-type) was the most selected, closely followed by a number of subdominant sequences, most of which did not represent miRNA seed sites (Figure 3D). Viruses with the let-7 seed (wild-type) had taken over the culture after two passages to naïve cells.

Finally, we randomized S1 in the context of an S2 let-7 seed site. After 120 hpe, the sequence UUUGUAG was the most selected followed by other sequences not representing miRNA seed sites (Figure 3C and D). Although UUUGUAG corresponds to the seed site for miR-1283, this miRNA is neither annotated for cow nor expressed in MDBK cells (35). The UUUGUAG sequence was the only one selected after two passages to naïve cells. To determine whether this specific sequence gave an advantage to the virus, we repeated the randomization experiment and again saw selection of miRNA unrelated sequences after passage to naïve cells (Supplementary Table S4). Although the most prevalent sequence, AUUUUGU, was not identical to the previously selected, both generated an AUUUUGUA motif, albeit 2 nt offset. Interestingly, let-7 was not among the top selected seeds. Finally, we randomized S1 in the context of an S2 miR-21 seed site. This RNA pool led to selection of the let-7 seed as the dominant sequence (data not shown).

In conclusion, while certain sequence motifs, including the let-7 seed site, may be preferred, miRNA binding to S1 does not appear to be critical. These experiments further emphasized that miRNA binding to S2 is important but also restricted to specific seeds of available miRNAs.

### miRNA binding remains important for viruses with other seed sites

**Figure 4.**
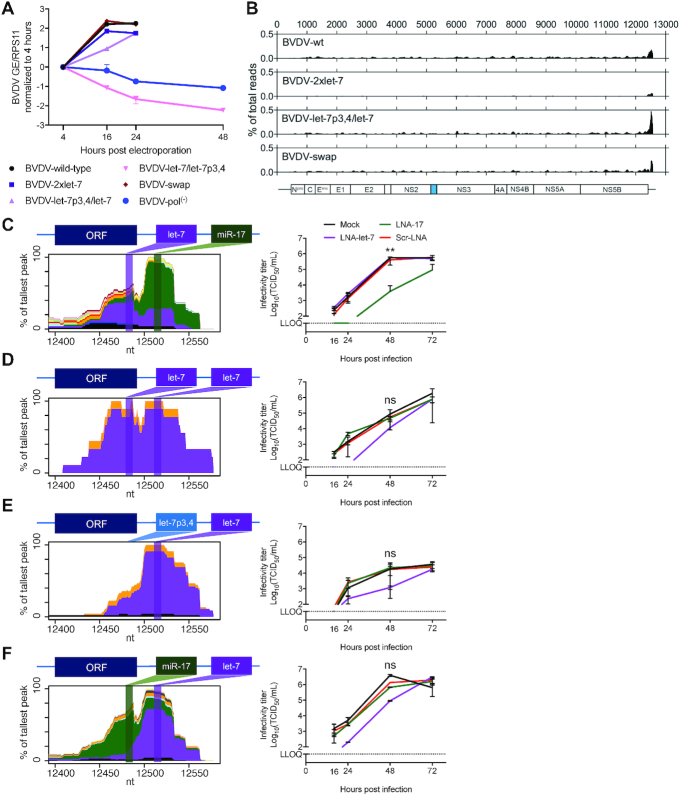
miRNA binding and dependence for BVDV variants with alternative seed sites. (**A**) Viral replication of BVDV-2×let-7, BVDV-swap and BVDV-S1p3,4 and BVDV-S2p3,4 mutants after electroporation of MBDK cells measured by RT-qPCR on intracellular RNA normalized to RPS11 and the 4 h time point. Data for BVDV wild-type and BVDV-pol^(−)^ are the same as in Figure 1B. (**B**) AGO/miRNA binding map across the genomes of BVDV seed site mutants normalized to library read depth. MDBK cells were infected (MOI = 0.1, except for BVDV-let-7p3,4/let-7 with MOI = 0.025) with the indicated mutants and, after 48 h, were cross-linked and processed for CLIP. Genome schematic with cellular Jiv90 insertion shown in cyan is drawn to scale below. (**C**–**F**) miRNA-specific binding maps and inhibitor experiments for BVDV seed site mutants. Left panels show miRNA-specific chimera-derived binding maps across the BVDV 3′ UTR seed site region. Schematics of BVDV genomes are depicted above each graph. The binding site locations for let-7 and miR-17 are shown across the graphs in purple and green shaded bars, respectively. Specific miRNA binding is color coded according to legend in Figure 2B. Right panels show infectious virus production, as measured by the TCID_50_ method after infection (MOI = 0.1) of LNA-treated MDBK cells. LLOQ: lower limit of quantification. Mean values (±SD), *n* = 3. Statistical differences by ANOVA to mock-treated BVDV (**C**–**F**) for the 48 h time point are shown. Corrections were done according to Dunnett’s test; ns, not significant; *P ≤ 0.05; **P ≤ 0.01; ***P ≤ 0.001.

Randomization experiments revealed alternative seed site selection at both S1 and S2. To determine whether the redirected mutants were functionally dependent on the respective miRNAs, BVDV-2×let-7, mutant versions of its individual seed sites (BVDV-let-7p3,4/let-7 and BVDV-let-7/let-7p3,4), and a swapped version of the wild-type seed site context (BVDV-miR-17/let-7, termed BVDV-swap) were engineered and electroporated into MDBK cells (see Supplementary Figure S2 for a complete list of mutants). BVDV-2×let-7 and BVDV-swap replicated to similar levels to BVDV wild-type (Figure 4A). BVDV-let-7p3,4/let-7 had slower kinetics, similarly to BVDV-let-7p3,4/miR-17 (Figure 1B), whereas no replication was detected for BVDV-let-7/let-7p3,4. No compensatory mutations or changes to the seeds were observed after sequencing this region.

To biochemically confirm miRNA binding, we performed CLEAR-CLIP, a modified version of AGO-CLIP that allows direct coupling of AGO-bound miRNAs to their cognate targets (28). Similar to BVDV wild-type, a major AGO/miRNA binding peak was detected at the end of the 3′ UTR for all other tested mutants (Figure 4B). Analysis of miRNA–target chimeras confirmed that BVDV wild-type bound let-7 and miR-17 to S1 and S2, respectively (Figure 4C). For BVDV-2×let-7, let-7 binding was observed at both sites (Figure 4D). BVDV-let-7p3,4/let-7 bound let-7 only to S2 (Figure 4E), whereas BVDV-swap bound miR-17 and let-7 to S1 and S2, respectively (Figure 4F).

To determine miRNA dependency of these viruses, we infected MDBK cells in the presence of LNA-17 or LNA-let-7. As expected, BVDV wild-type produced ∼100-fold lower titers under LNA-17 inhibition but was unaffected by LNA-let-7 and the control LNA. BVDV-2×let-7, BVDV-let-7p3,4/let-7 and BVDV-swap exhibited 10–100-fold lower titers under LNA-let-7 treatment compared to other conditions (Figure 4C-F). However, even with the highest non-cytotoxic LNA-let-7 concentration, complete let-7 inhibition could not be achieved in MDBK cells (22); thus, the effect of let-7 inhibition may be underestimated.

These results demonstrate that mutant viruses bound and depended on the corresponding miRNAs. S2 was further shown to be more important compared to S1, regardless of whether the seed site for S2 is recognized by miR-17 or let-7. Nevertheless, S1 appears to be important in certain contexts, as for BVDV-let-7p3,4/let-7, which generally exhibited 10-fold lower titers compared to other viruses.

### Most selected seed site variants are highly viable

**Figure 5.**
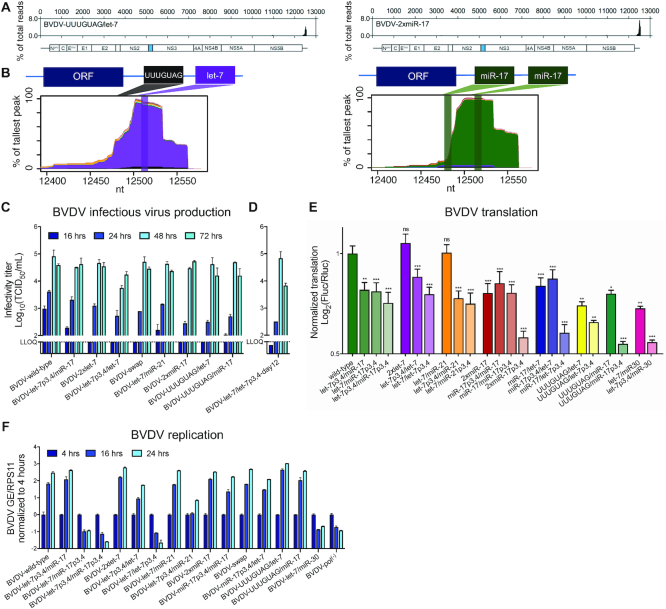
Infection kinetics and translation efficiencies of BVDV seed site mutants. AGO/miRNA binding maps across the genome (**A**) and miRNA-specific chimera-derived binding profiles across the 3′ UTR seed site regions (**B**) are shown for BVDV-UUUGUAG/let-7 and BVDV-2×miR-17. MDBK cells were infected (MOI = 1) with the indicated mutants and after 24 h cells were cross-linked and processed for CLIP. Annotation as in Figure 4. (**C**) Infectious virus production (TCID_50_/ml) after infection (MOI = 0.01) of MDBK cells with third passage stocks of BVDV seed site mutants. (**D**) Virus production as in (**C**) with third passage stock of a culture adapted population of viruses that came from a BVDV-let-7/let-7p3,4 clone propagated for 12 days (BVDV-let-7/let-7p3,4-day12) in MDBK cells. LLOQ: lower limit of quantification. Error bars represent SD of the mean. (**E**) BVDV IRES-driven translation measured by bidirectional reporters with and without modification of the S1 and S2 miRNA seed sites. Translation levels from all reporters were normalized to the wild-type and differences to this were compared by ANOVA analysis. Corrections were done according to Dunnett’s test; ns, not significant; *P ≤ 0.05; **P ≤ 0.01; ***P ≤ 0.001. Mean values (±SEM), *n* = 3. (**F**) Viral replication of BVDV-S1p3,4 and BVDV-S2p3,4 mutants after electroporation of MDBK cells measured by RT-qPCR on intracellular RNA normalized to RPS11 and the 4 h time point. Data for BVDV-miR-30 and all S2p3,4 mutants (except for BVDV-let-7p3,4/miR-17p3,4) are the same as in Figures 1B and 4A. Mean values (±SD), *n* = 2.

Since we observed attenuated replication and virus production for BVDV-let-7p3,4/let-7, we wanted to directly compare infection kinetics of the different BVDV mutants. Other variants identified in randomization experiments, BVDV-2×miR-17, BVDV-let-7/miR-21, BVDV-UUUGUAG/let-7 and BVDV-UUUGUAG/miR-17, were also constructed; these caused CPEs in cell culture 2–4 dpe. No changes to the seed sites of these viruses were observed. Using AGO-CLIP experiments, we confirmed absence of miRNA binding to S1 and let-7 binding to S2 for BVDV-UUUGUAG/let-7 (Figure 5A and B). Unlike BVDV-2×let-7 and BVDV-swap, no indication of miRNA binding to S1 was evident in repeated experiments for BVDV-2×miR-17 (Figure 5A and B). This emphasized the importance of seed site context for miRNA binding to the BVDV 3′ UTR. In comparative kinetic experiments using third passage virus stocks, BVDV wild-type was the most fit with ∼10-fold higher infectivity titers compared to the mutants at early time points (Figure 5C). Most mutants, however, produced titers similar to BVDV wild-type 48 h post-infection (hpi). Only BVDV-let-7p3,4/let-7 had markedly slower kinetics. Kinetic data therefore suggested that BVDV can retain high viability even in the absence of S1 let-7 and S2 miR-17 binding.

Since unaffected or only slightly attenuated viability was observed, we looked for compensatory mutations elsewhere in the viral genome. After three passages in cells, BVDV wild-type and most mutants had acquired mutations in the ORF, some of which were common (Supplementary Table S5). BVDV-UUUGUAG/let-7 did not acquire any mutations. BVDV-let-7p3,4/let-7, which was the most attenuated variant, acquired the most mutations suggesting a larger pressure on this virus to increase its fitness. For several variants, including BVDV wild-type, the five 3′ terminal C residues were reduced to four, likely reflecting variability at the very end of the BVDV genome unrelated to seed site mutations. Therefore, no hotspots of seed site compensatory mutations were identified.

### The S1 let-7 site is important for full translational efficiency

Stimulation of translation by miR-122 was previously demonstrated for HCV (12), and for related equine (26,36) and bovine hepaciviruses (15). For BVDV, a similar role was shown for miR-17 and let-7 (22). To study the effect of seed site variation on translation, we cloned the seed sites of the variants described above and corresponding S1/S2 mutations (p3,4) into a bidirectional reporter (Supplementary Figure S3). For the wild-type reporter, mutation of either S1 or S2 led to equal translation reduction, whereas combined mutation led to further reduction of translation (Figure 5E). Upon comparing all seed site mutants, full translational efficiency was only observed in the context of S2 seeds of viable mutants (i.e. miR-17, let-7 and miR-21) harboring a let-7 seed at S1. Combinations with other S1 seeds (i.e. miR-17 and UUUGUAG) led to translation levels in the range of let-7p3,4/miR-17p3,4, while further reduction occurred upon mutation of S2. For the let-7/miR-30 context, corresponding to a non-replicating virus, translation was comparable to let-7p3,4/miR-17p3,4 and was further reduced upon S1 mutation. Altogether, our results suggested that the combination of a seed corresponding to an available miRNA at S2 and the presence of a let-7 seed at S1 is necessary for full translational efficiency.

To delineate whether translation levels were coupled to replication levels, we engineered and electroporated full BVDV genomes corresponding to the tested mutants (Figure 5F). As expected, BVDV-let-7/miR-30 and mutants harboring S2p3,4 mutations did not have detectable levels of replication. Furthermore, no clear correlation was observed between efficiency of translation and initial viral replication levels of the mutants. This suggested that the minor effect on translation (60–80% of wild-type) is not associated with a significant effect on the overall replication level.

### Acquisition of a short sequence at the 3′ terminus allows miRNA independence

**Figure 6.**
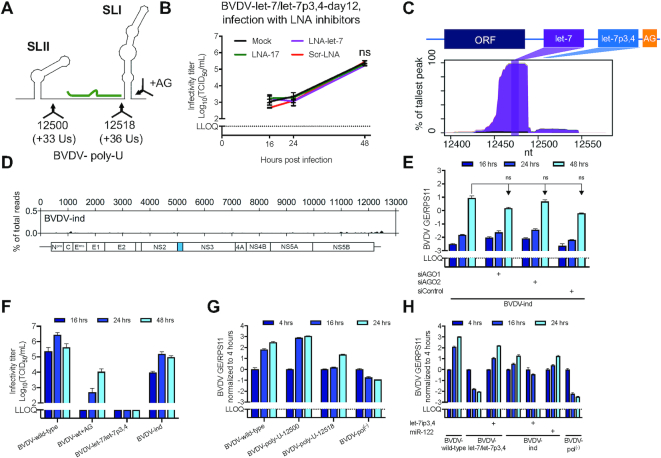
BVDV modifications enabling miRNA-independent replication. (**A**) Schematic of the BVDV wild-type genomic 3′ end depicting the location of the AG and poly-U insertions. (**B**) Infectious virus production (TCID_50_/ml) after infection (MOI = 0.1) with BVDV-let-7/let-7p3,4-day12 supernatant of LNA-treated MDBK cells. Schematic of the representative BVDV genome is given in (**C**). miRNA-specific chimera-derived binding profiles across the 3′ UTR seed site region (**C**) and AGO/miRNA binding maps across the genome (**D**) for the engineered BVDV-independent mutant, BVDV-ind. MDBK cells were infected (MOI = 1), and after 24 h cells were cross-linked and processed for CLIP. Annotation as in Figure 4. (**E**) Viral replication of BVDV-ind in AGO-depleted MDBK cells after infection (MOI = 0.01) measured by RT-qPCR on intracellular RNA normalized to RPS11 and the 4 h time point. (**F**) Infectious virus production (TCID_50_/ml) in MDBK cells after electroporation with AG insertion variants. (**G**) Viral replication of BVDV-poly-U variants after electroporation of MDBK cells measured by RT-qPCR on intracellular RNA normalized to RPS11 and the 4 h time point. (**H**) Viral replication of BVDV-let-7/let-7p3,4+AG insertion variants after electroporation of MDBK cells measured by RT-qPCR on intracellular RNA normalized to RPS11 and the 4 h time point. Trans-complementation with miRNA mimics at 10 nM is indicated. LLOQ: lower limit of quantification. Mean values (±SD), *n* = 2. Statistical differences to mock (**B**, **E**) were analyzed using ANOVA. Results for the last time points (**E**) are shown; corrections were done according to Dunnett’s test; ns, not significant; *P ≤ 0.05; **P ≤ 0.01; ***P ≤ 0.001.

HCV miR-122-independent variants were previously described (24,25). To identify potential miRNA-independent BVDV variants, we monitored cells infected with BVDV-let-7/let-7p3,4 and BVDV-miR-17/miR-17p3,4, which were non-viable in short-term experiments, for a longer time frame. Interestingly, we observed CPEs at 12 and 14 dpe, respectively. Whereas seed site sequencing revealed that the latter had reverted to BVDV-2×miR-17, no seed site changes were observed for the BVDV-let-7/let-7p3,4-day12 variant. Despite the lack of a functional S2, virus production for the BVDV-let-7/let-7p3,4-day12 variant was surprisingly comparable to most other tested variants (Figure 5D). We therefore did full-genome sequencing and identified duplication of an AG dinucleotide motif at positions 12572–73, separated from the genomic 3′ terminus only by the four to five terminal Cs (Figure 6A and Supplementary Table S5). Analysis of individual clones identified the AG insertion for eight of nine clones, while four of nine had deleted one to two of the terminal Cs or replaced them with Us (Table 1). Interestingly, the BVDV-let-7/let-7p3,4-day12 virus was unaffected by the presence of LNA-17 or LNA-let-7 (Figure 6B). We therefore engineered the original BVDV-let-7/let-7p3,4 mutant with the AG insertion to create BVDV-let-7/let-7p3,4+AG (BVDV-ind hereafter). Unlike other mutants analyzed, no AGO/miRNA binding peak was observed at S2 after AGO-CLIP analysis, whereas a let-7 peak was retained at S1 (Figure 6C and D). Furthermore, BVDV-ind replicated independently of AGO1 and AGO2 (Figure 6E). Altogether, these data suggested a mechanism of miRNA-independent replication associated with sequence modification at the terminus of BVDV 3′ UTR.

**Table 1.** Summary of mutations in the BVDV 3′ terminal region from experiments on miRNA independence.

BVDV genome	Engineered sequence	S2 reversion	Clones	3′ terminus
BVDV-WT	UAACAGCCCCC		N/A	UAACAGCCCC(C)
BVDV-WT+AG	UAACAGAGCCCCC		N/A	UAACAG***__***CCCC
BVDV-let-7/let-7p3,4+AG	UAACAGAGCCCCC	No	3	UAACAGAGCCCC
			1	UAACAGAGCCC ***_***
			1	UAACAGAGCCC***U***
			1	UAACAGAGCC***U***C
			1	UAACAGAGCC***UU***
			1	UAACAGAGCC***UUU***
			1	UAACAGAC***___***
BVDV-let-7/let-7p3,4+AG+mimic	UAACAGAGCCCCC	No	N/A	UAACAG***__U***CCC
BVDV-WT+2N	UAACAGNNCCCCC		10	UAACAG__CCCCC
BVDV-let-7/let-7p3,4+2N	UAACAGNNCCCCC	No	7	UAACAGAGCCC***_***
			2	UAACAGAGCC***G_***
			1	UAACAGAGC***A***C***_***
BVDV-let-7/miR-17p3,4+2N	UAACAGNNCCCCC	Yes	6	UAACAG__C***A***CCC
			1	UAACAG__CCCCC
			1	UAACAG__C***A***CC
			1	UAACAG__C***A***C***UU***
			1	UAACAG__C***A***C***U***
BVDV-miR-17/miR-17p3,4+2N first passage	UAACAGNNCCCCC	No	4	UAAUAA***UUU***C***U***C
		No	4	UAACAG__CCCC***U***
		Yes	1	UAACAG**__**CCCCC***CU***
		Yes	1	UAACAG**__**CCCC
BVDV-miR-17/miR-17p3,4+2N second passage	UAACAGNNCCCCC	Yes	4	UAACAG__CCCC
BVDV-miR-17/miR-17p3,4-UAAUUUCUC	UAAUAAUUUCUC	No	2	UAAUAAU***__***C***C***C
			2	UAAUAAU***__***C***__***
			1	UAAUAAUU***____***
			1	UAAUAAUUU***_U_***
			1	UAAUAAUU***AU***U***_***
			1	UAAUAAUU***_***CU***_***
			1	UAAUAAU***CC***CU***_***
			1	UAAUAAUU***C***UC***AUAG***

Analysis was done for individual bacterial clones acquired after purifying and cloning RNA from supernatant passaged twice to naïve cells unless otherwise indicated. Engineered sequences are underlined to discriminate from the parental sequence and resulting changes are bold italicized. Deletions of the engineered or the consensus sequence are indicated by underscores. The number of bacteria clones that had the reported sequence are indicated, except for cases of bulk sequencing (N/A).

To determine whether the identified AG insertion specifically rescued replication of the BVDV-let-7/let-7p3,4 mutant, we engineered the insertion in the wild-type context to create BVDV-wt+AG. BVDV-ind produced ∼10-fold and BVDV-wt+AG ∼100-fold lower titers than BVDV wild-type, respectively (Figure 6F). Sequencing after passaging to naïve cells showed that BVDV-wt+AG had deleted the inserted dinucleotide sequence, while no changes occurred to the wild-type and BVDV-ind 3′ UTRs. This demonstrated that insertion of the AG dinucleotide at position 12574 in the 3′ UTR indeed rescued BVDV-let-7/let-7p3,4 virus production, whereas it was not tolerated in the wild-type context.

### Cross-talk between the S2 region and the 3′ terminal sequence is required for BVDV replication

As the location and spacing of S1 and S2 are highly conserved among typical pestiviruses (22), we examined whether the distance between the two seeds and the 3′ terminus is important. We therefore inserted spacer sequences immediately up- or downstream of S2 in BVDV wild-type. BVDV-poly-U-12500, similarly to the recently described CSFV Piñar del Rio strain (37), carried a 36-nt poly-U stretch (33 Us inserted) upstream of the miR-17 site (Figure 6A). This virus was highly fit causing complete CPEs 2 dpe and had comparable replication levels to BVDV wild-type (Figure 6G). Sequencing of the 3′ UTR showed that it retained the poly-U tract after passage to naïve cells. This suggested that neither the distance between S1 and S2 nor direct interaction between S2 and the upstream sequence is critical for replication. BVDV-poly-U-12518, carrying a 36-nt poly-U tract insertion immediately downstream of S2, on the contrary, was delayed compared to BVDV wild-type causing CPEs 5 dpe, and had precisely reverted to wild-type after passage to naïve cells (Figure 6A and G). In another replicate experiment, deletion left one U replacing the A present at position 1 of the miR-17 seed (GCACUUUU). This suggested that insertion is not tolerated between S2 and the 3′ terminal sequence and that this sequence context may function as an entity. Combined with the location of the adaptive changes responsible for miRNA independence, these results indicate that a close cross-talk between the S2 region and the 3′ terminal sequence is required for BVDV replication.

### miRNA binding and 3′ end modification are mutually exclusive mechanisms supporting BVDV replication

To confirm whether miRNA binding and AG insertion contribute to mutually exclusive mechanisms to stimulate BVDV replication and virus production, we trans-complemented BVDV-let-7/let-7p3,4 mutants with let-7i-p3,4 mimic. Trans-complementation rescued BVDV-let-7/let-7p3,4 replication to almost wild-type levels, whereas BVDV-ind replication interestingly was attenuated (Figure 6H). Sequencing of the trans-complemented cultures after two passages showed that the S2 site was retained for both, while BVDV-ind had changed the terminal CAGAGCCCCC sequence to CAGUCCC, thereby deleting the duplicate AG sequence. That result was consistent with the delay and reversion observed for the BVDV-wt+AG mutant (Figure 6F). Thus, S2 miRNA binding and compensatory 3′ end mutagenesis appear to be mutually exclusive ways to control BVDV replication.

### 3′ terminal sequence modification is a general way of compensating for miRNA binding

To finally address whether this mechanism of miRNA independence was exclusive to the let-7/let-7p3,4+AG combination, we engineered dinucleotide insertions in the wild-type, let-7/let-7p3,4, let-7/miR-17p3,4 and miR-17/miR-17p3,4 contexts. To allow alternative 3′ end adaptation, we randomized the dinucleotide insertion to generate 2N variants. Electroporation led to complete CPEs for the BVDV-wild-type+2N and BVDV-let-7/let-7p3,4+2N experiments 3–4 dpe. For the BVDV-let-7/miR-17p3,4+2N and BVDV-miR-17/miR-17p3,4+2N experiments, CPEs were observed after 12 days in culture. Analysis after the second passage of BVDV-WT+2N and BVDV-let-7/miR-17p3,4+2N revealed that most had reverted to the wild-type genotype with the exception of some clones containing single nucleotide differences at the antepenultimate position of the genomic terminus (Table 1). All BVDV-let-7/let-7p3,4+2N clones had selected the AG dinucleotide without reversion of the seed site, demonstrating a specific advantage of AG over other dinucleotide insertions. All BVDV-miR-17/miR-17p3,4+2N clones had reverted to BVDV-2×miR-17. However, 6 of 10 sequenced clones after the first passage retained the S2p3,4 mutations and had acquired the alternative 3′ terminus UAAUUUCUC instead of CAGCCCCC. Electroporation of the engineered BVDV-miR-17/miR-17p3,4-UAAUUUCUC mutant led to visible CPEs 5 dpe and complete cell death 10 dpe. Analysis of 10 clones after the second passage showed that all had retained the S2p3,4 mutations but further adapted their 3′ termini to the sequence UAA(Y)_2–5_ (Table 1). In fact, this virus also duplicated a short nucleotide motif, in this instance the UAA trinucleotide present at position 12568–70. Modification of the BVDV genomic 3′ terminus therefore appears to be a general way of compensating for the absence of miRNA binding.

## DISCUSSION

In this study, we interrogated dependence and tropism of BVDV on host-derived miRNAs. We showed that miR-17 binding to S2 is important for translation and is indispensable for replication and virus production. Interestingly, like HCV (11), BVDV requires association with AGO2 to replicate. We further demonstrated that auxiliary pairing provides limited stimulation of replication. Therefore, and in contrast to HCV (38), only the miRNA interaction with the seed region seems critical for BVDV replication. MiRNA antagonism or mutation of the S1 let-7 site led to only minor attenuation of replication and virus production. Whereas this was consistent with only minimal inhibition by let-7 antagonism in our previous study, more severe attenuation of the BVDV-S1p3,4 mutant was then observed (22). This difference may be ascribed to nucleotide differences between the parental clones used in the two studies. A functional let-7 site was nonetheless important for full translational efficiency; however, this did not directly translate to differences in replication. Therefore, S1 miRNA binding and regulation of viral translation seem to play an auxiliary role for the life cycle of BVDV.

HCV miRNA tropism can be redirected to non-liver-specific miRNAs through mutagenesis or seed site randomization (13,34). This only allows marginal replication in non-liver cells though, suggesting that other liver-specific factors contribute to HCV tissue specificity. Here, we used seed site randomization and deep sequencing as a powerful evolutionary tool to allow viral selection of different viable combinations of alternative sequences. In an environment with unrestricted access to miR-17, BVDV promptly selected miR-17, demonstrating a potent tropism for this miRNA acquired during natural evolution of S2. Under miR-17 inhibition, however, BVDV S2 tropism could be redirected to let-7 or miR-21, both among miRNAs highly expressed in MDBK cells and with broad tissue distribution. Interestingly, other abundant miRNAs such as the miR-30 family were not selected, although supplementation with miR-30d mimic allowed the BVDV-miR-30 mutant to efficiently replicate. It would therefore appear that either miR-30 was not accessible at the site of replication, presumably saturated while bound by its natural mRNA targets, or its abundance was overestimated in our assay. S1, on the other hand, could be substituted for a number of non-miRNA-related sequence combinations. Although let-7 was among the top selected seed sites, it therefore appears that constraints were not as strict as for S2, further highlighting the lower importance of S1 compared to S2. In randomization experiments, we also observed that BVDV-2×miR-17 eventually outcompeted BVDV-swap. Therefore, miR-17 binding to S2 regardless of S1 interaction appears to give an overall advantage to BVDV. We conclude that BVDV has a potent, evolutionary acquired tropism for miR-17 at S2, which can experimentally be redirected to other available miRNAs.

Why BVDV evolved to specifically bind miR-17 at S2 is intriguing. In our *in vitro* studies, BVDV-2×let-7 produced infectivity titers similar to BVDV wild-type, and similarly to miR-17, let-7 is broadly expressed across tissue types (23). Becoming dependent on a pro-proliferatory miRNA like miR-17 (39) might be advantageous to pestiviruses as they could thereby take advantage of the metabolism of dividing cells (40). We previously showed that, during infection, binding by BVDV S2 can sequester enough miR-17 to act as a sponge to functionally derepress miR-17 targeted mRNAs, whereas this is not the case for let-7 binding to S1 (22). Therefore, an alternative hypothesis could be that sequestration of a pro-proliferatory miRNA like miR-17 may be, for example, beneficial for the virus in immune cells since this would dampen proliferation of antigen-presenting cells. Whether miRNA sponging plays a functional role for any virus during infection of its natural host organism remains to be demonstrated. Studying infection kinetics and tissue tropism of mutants that have altered or no miRNA dependency in animal models should therefore provide further insights into the effect of BVDV on the miRNA–mRNA host interactome.

RNA structures as well as essential and non-essential regions in the BVDV 3′ UTR were previously defined (3,41,42). Although viral regulation by miRNAs was an uncharted area at the time, mutagenesis across the miR-17 site led to inhibition of replication, whereas deletion of the miR-17 auxiliary pairing region or the let-7 site led to only minor attenuation (3,41). This was supported by our current findings. It was further shown that upstream stem loops were partially dispensable for replication, whereas S2-deleted viruses could not be recovered in these studies. To further understand the sequence constraints on the S2 region, we inserted poly-U tracts up- or downstream of S2. In culture, these resulted in rapid and precise deletion of the inserted downstream sequence, while the upstream insertion was tolerated. It thus appears that both spacing and interaction between S2 and SLI are important for the viral life cycle. Cross-talk between S2 and the 3′ terminus was further supported by the finding that the BVDV-let-7/let-7p3,4 mutant became miRNA independent through acquisition of compensatory mutations close to the 3′ terminus. However, the same set of mutations was not permitted in context of the BVDV wild-type. Along with trans-complementation data for BVDV-ind, it was clear that modification of the 3′ terminus is a compensatory mechanism that is mutually exclusive with miRNA binding at S2. Finally, 3′ end mutagenesis seems to be a general mechanism compensating for the inability of the virus to bind miRNAs as another short sequence duplication allowed replication of a mutant with an miR-17/miR-17p3,4 seed site context. Remarkably, in both instances the duplicated motifs were situated at closely spaced positions of SLI. Related to these observations, HCV-escape variants to miR-122 antagonism were observed outside of the miRNA seed sites and very close to the 5′ terminus (43), therefore suggesting a similar although mirrored mechanism of miRNA escape.

**Figure 7.**
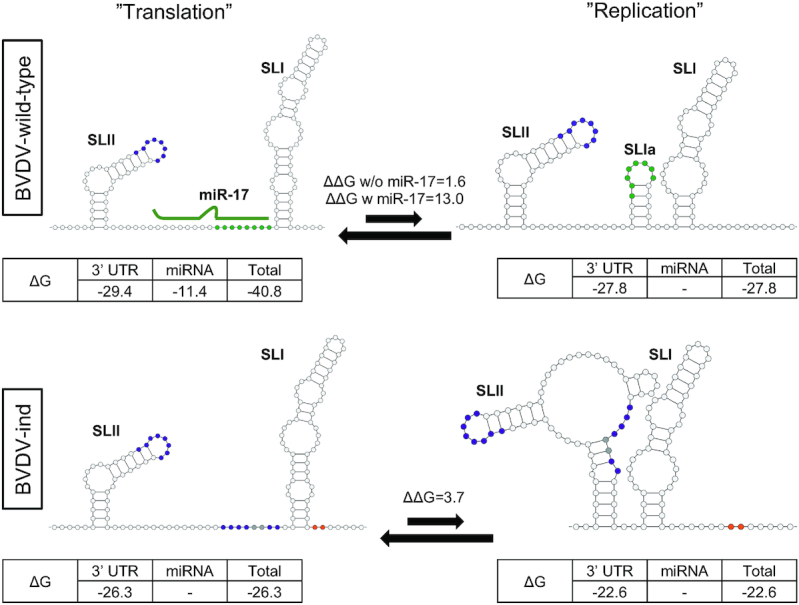
Putative model for miRNA-mediated structure switching. Predicted structure alternatives for BVDV wild-type with and without miR-17 binding (top) and BVDV-ind (bottom) are shown including folding energies (kcal/mol). Only S2 binding was assumed. miR-17 seed sites are drawn in green, let-7 in purple, p3,4 mutations in gray and 3′ UTR insertions in orange. Arrows represent the balance between structural states and the difference in energy (ΔΔ*G*) is given.

For HCV, mutagenesis of each seed site independently led to abrogation of RNA translation and replication, but some studies reported a stronger dependence on S1 (38,44–46). Here, we demonstrate that only S2 is critical for BVDV replication and virus production. Therefore, the seed site most proximate to the genomic termini (S1 for HCV and S2 for BVDV) appears to be most important. It was recently suggested that miR-122 binding allows for stabilization of the HCV IRES and thereby blocks formation of alternative IRES-disrupting structures while still allowing alternative replication mediating structures on the negative strand (32,47). Modifications of the BVDV 3′ end may thus similarly impact the folding energy of RNA structures in ways that compensate for miRNA interactions. Both S2 and the acquired 3′ sequences are adjacent to the base of the terminal SLI, which is close to the initiation site for negative-strand RNA synthesis. Predictions show that binding of miR-17 to S2 of BVDV wild-type would stabilize a structure containing a long terminal SLI, which is the structure that was previously experimentally determined (3,41,42,48). This would further prevent formation of an alternative structure forming a smaller SLIa across the S2 region, which would cause SLI to partly unfold releasing a longer single-stranded 3′ tail (Figure 7). Release of the 3′ end could potentially provide an initiation site for negative-strand synthesis. That formation would be desired for a smaller fraction of the RNA molecules, reflected by its less favorable energetic state, as most viral RNA would be required to engage in viral protein production. Thus, miRNA binding could specifically be needed by BVDV wild-type to obtain the right balance (Figure 7). BVDV-let-7/let-7p3,4, on the other hand, is not predicted to form an alternative structure with a longer single-stranded 3′ tail unless it acquires the AG insertion (Supplementary Figure S4). For BVDV-ind, the alternative structure is energetically less favorable compared to BVDV wild-type (ΔΔ*G* = 3.7 versus ΔΔ*G* = 1.6), possibly explaining why miRNA stabilization is not necessary (Figure 7). For BVDV-poly-U-12518, the alternative structure would not form, providing a possible explanation to why this mutant is non-viable. Arguing against this hypothesis, however, is the fact that a BVDV-S2p7,8 mutant was efficiently rescued by miR-17p7,8 mimic although these mutations would obliviate formation of putative SLIa and thereby 3′ end liberation (22). Thus, if the alternative structure including SLIa does form, it is non-essential. Similarly, BVDV-2×let-7, which replicates to wild-type levels, is not predicted to form the alternative structure. The mechanism of BVDV miRNA regulation, therefore, still needs to be further investigated. Application of high-throughput techniques to map RNA structures may uncover additional details. One proposed mechanism of HCV/miR-122 interaction suggests competition between miR-122 and cellular proteins, such as poly-C binding protein 2, for binding the HCV genome, thereby providing a switch between translation and replication (49). For BVDV, recruitment of NFAR family proteins was also suggested to regulate these mechanisms, potentially by interacting with both UTRs (48). Therefore, alternatively to structural switching, interaction or competition of other host factors with miR-17 could fine-tune the balance of viral RNA engagement in translation versus replication. This could potentially be investigated using high-throughput RNA interaction methods (50,51).

In conclusion, we have characterized the functional requirements of BVDV–miRNA interactions. We have demonstrated that BVDV miRNA tropism can be redirected to alternative host miRNAs. Interestingly, variants able to replicate independently of miRNAs through 3′ terminal compensatory mutations were identified. Therefore, BVDV provides a versatile platform to study miRNA biology as it exists in cp and ncp formats, infects a plethora of tissues and can replicate utilizing any or even no miRNAs. It remains to be seen whether other virus families depend on host miRNAs. It appears that pestiviruses are capable of miRNA-independent replication, but that advantages of miRNA dependence either for direct replication purposes or for tropism strategies during evolutionary adaptation led them to maintain miR-17 dependency.

## DATA AVAILABILITY

Data processing for deep sequencing and AGO-CLIP was done in Galaxy (usegalaxy.org) and R (r-project.org) and structural predictions with Mfold (unafold.rna.albany.edu), VARNA (varna.lri.fr/), IntaRNA (http://rna.informatik.uni-freiburg.de/IntaRNA/Input.jsp) and ViennaRNA Web Services (http://rna.tbi.univie.ac.at). AGO-CLIP data have been uploaded to the publicly available library GEO under accession GSE146910.

## Supplementary Material

gkaa300_Supplemental_Files
