# Supplementary material for: Evolutionary selection of pestivirus variants with altered or no microRNA dependency
Source: Nucleic Acids Res. 2020 May 6;48(10):5555–71. doi: 10.1093/nar/gkaa300 (PMC7261151; doi:10.1093/nar/gkaa300)
Supplement: gkaa300_Supplemental_Files [file gkaa300_supplemental_files.zip › Kokkonos - BVDV miRNA tropism Supplementaries revised FINAL proofs.docx]

**SUPPLEMENTARY DATA**

**Evolutionary selection of pestivirus variants with altered or no microRNA dependency**

Konstantinos G. Kokkonos^1^, Nicolas Fossat^1^, Louise Nielsen^1^, Christina Holm^1^, Wytske M. Hepkema^1^, Jens Bukh^1^ and Troels K. H. Scheel^1,2,*^

^1^ Copenhagen Hepatitis C Program (CO-HEP), Department of Infectious Diseases, Hvidovre Hospital, Hvidovre, 2650, Denmark and Department of Immunology and Microbiology, Faculty of Health and Medical Sciences, University of Copenhagen, Copenhagen, 2200, Denmark.

^2^ Laboratory of Virology and Infectious Disease, Center for the study of Hepatitis C, The Rockefeller University, New York, NY, 10065, USA.

* Troels K. H. Scheel. Tel: +45 25 122 771; Fax: +45 35 32 78 74; Email: tscheel@sund.ku.dk

**SUPPLEMENTARY TABLES**

**Table S1:** Summary of reverse primers and templates used to generate the randomized BVDV libraries. In all PCRs the same forward primer was used which started with the T7 promoter (TS-O-00122).

| **BVDV genome** | **Primer name** | **Template** |
| --- | --- | --- |
| BVDV-let-7/7N | TS-O-00212 | BVDV-wild-type |
| BVDV-miR-17/7N | TS-O-00212 | BVDV-miR-17/miR-17p3,4 |
| BVDV-7N/miR-17 | TS-O-00229 | BVDV-let-7/miR-17p7,8 |
| BVDV-7N/let-7 | TS-O-00548 | BVDV-let-7/miR-17p7,8 |
| BVDV-wt+2N | TS-O-01077 | BVDV-wt+AG |
| BVDV-let-7/let-7p3,4+2N | TS-O-01077 | BVDV-let-7/let-7p3,4 |
| BVDV-let-7/miR-17p3,4+2N | TS-O-01077 | BVDV-let-7/miR-17p3,4 |
| BVDV-miR-17/miR-17p3,4+2N | TS-O-01077 | BVDV-miR-17/miR-17p3,4 |

**Table S2:** Primers used for experimental procedures.

| **Primer name** | **Primer sequence** |
| --- | --- |
| 1. **Degenerate primers for BVDV randomization** | |
| TS-O-00212 (Rev) | GGGGGCTGTTAGAGGTCTTCCCTAGTCCAACTATAGACGTCGGGCGTATCCTCATACAG  CT**NNNNNNN**TGTGTGCATTAAATG |
| TS-O-00229 (Rev) | GGGGGCTGTTAGAGGTCTTCCCTAGTCCAACTATAGACGTCGGGCGTATCCTCATACAG  CTAAAGTGCTGTGTGCATTAAATGTAGTGTTATCTT**NNNNNNN**ATAATCTTGACTAC |
| TS-O-00548 (Rev) | GGGGGCTGTTAGAGGTCTTCCCTAGTCCAACTATAGACGTCGGGCGTATCCTCATACA  GCTGAGGTAGTGTGTGCATTAAATGTAGTGTTATCTT**NNNNNNN**ATAATCTTGACTAC |
| TS-O-01077 (Rev) | GGGG**NN**CTGTTAGAGGTCTTCCCTAGTCCAACTATAGACGTC |
| TS-O-01097 (Rev) | GGGGG**NN**CTGTTAGAGGTCTTCCCTAGTCCAACTATAGACGTCGGGCGTATCCTCATAC  AGCTACGTTATTGTGTGCATTAAATG |
| 1. **T7 promoter addition for long PCRs** | |
| TS-O-00122 (Fwd) | GCTAGATAATACGACTCACTATAGTATACGAGAATTAGAAAAGGCAC |
| 1. **qPCR primers/probes** | |
| TS-O-00061 (Fwd) | GGTGACTGCAGGTCGGGTA |
| TS-O-00062 (Rev) | GGTAAAATAGTGGCCCTGGCTT |
| TS-O-00063 (Fwd) | /FAM/CAGAGGACCTGTGAGCGGGATCTACCT/BHQ/ |
| TS-O-00094 (Fwd) | GCCGAGACTACCTTCACTAC |
| TS-O-00095 (Rev) | AGGTCCAGTCTCAGAACTTC |
| TS-O-00096 (Fwd) | Cy5/CACAAGAACATGTCCGTGCACCTTTCT/BHQ-2 |
| 1. **cDNA production** | |
| TS-O-00036 (For) | GTCCCTGTTAGGTGGTCCGAC |
| TS-O-00058 (Rev) | GGCTGTTAGAGGTCTTCCC |
| 1. **Deep sequencing** | |
| TS-O-00390 | AATGATACGGCGACCACCGAGATCTACACTATAGCCTACACTCTTTCCCTACACGACGC |
| TS-O-00391 | CAAGCAGAAGACGGCATACGAGATCGAGTAATGTGACTGGAGTTCAGACGTGTGC |
| TS-O-00392 | AATGATACGGCGACCACCGAGATCTACACATAGAGGCACACTCTTTCCCTACACGACGC |
| TS-O-00393 | CAAGCAGAAGACGGCATACGAGATTCTCCGGAGTGACTGGAGTTCAGACGTGTGC |
| TS-O-00394  (Fwd) | CACTCTTTCCCTACACGACGCTCTTCCGATCTCATAGTCAATCTGCTGCTGAG |
| TS-O-00395  (Rev) | GACTGGAGTTCAGACGTGTGCTCTTCCGATCTGGCTGTTAGAGGTCTTCCCT |
| 1. **Full-ORF amplification** | |
| TS-O-00787  (Fwd) | TAATACGACTCACTATAGTATACGAGAATTAGAAAAGGCAC |
| TS-O-00788  (Rev) | GTTAGAGGTCTTCCCTAGTCCAACTA |
| 1. **5’ end determination** | |
| TS-O-00038  (Rev) | CCTCTGCTATTACCCGAC |
| TS-O-00039  (Rev) | TCCTCACCTGGTATTTGACTC |
| TS-O-01059  (Rev) | GTCTGAGCAACTTGTGACCCATAGAGGGC |
| 1. **3’ end determination** | |
| TS-O-00913  (Rev) | GGAAAGCATTATGAGCAACTGCAGCTA |
| TS-O-00915  (Rev) | ATACAAGTTAGGTCCCATAG |
| AUAP | GGCCACGCGTCGACTAGTAC |

**Table S3:** Summary of siRNA, small RNA and LNA oligo sequences used in this study. For LNAs, + in front of a nucleotide indicates LNA, and * after a nucleotide indicates DNA phosphorothioate backbone.

| **LNA/mimic/siRNA** | **Sequence** |
| --- | --- |
| Tiny-LNA-17 | +A+G+C+A+C+T+T+T |
| Tiny-LNA-let-7 | +A+C+T+A+C+C+T+C |
| Scrambled LNA | +TC*+AT*A*+C+TA*T*+AT*+GA*+C+A |
| miR-17 | CAAAGUGCUUACAGUGCAGGUAG |
| miR-17p3,4 | CAUUGUGCUUACAGUGCAGGUAG |
| miR-20a p3,4 | UAUUGUGCUUAUAGUGCAGGUAG |
| miR-93p3,4 | CAUUGUGCUGUUCGUGCAGGUAG |
| miR-106b p3,4 | UAUUGUGCUGACAGUGCAGAU |
| miR-122 | UGGAGUGUGACAAUGGUGUUUG |
| miR-17p3,4,14-16 | CAUUGUGCUUACACACCAGGUAG |
| miR-17p3,4,21-23 | CAUUGUGCUUACAGUGCAGGAUC |
| miR-17p3,4,19,20 | CAUUGUGCUUACAGUGCACCUAG |
| miR-30d | UGUAAACAUCCCCGACUGGAAGCU |
| let-7i-p3,4 | UGUCGUAGUAGUUUGUGCUGUU |
| siAGO1_1_ | GAGAAGAAGUGCUCAAGAAUU |
| siAGO2_1_ | GCACGGAGGUGCACCUCAAUU |
| siAGO2_2_ | GCAGGACAGAGAUGCAUAAUU |
| siGENOME Non-Targeting siRNA #4 | Horizon Cat # D-001210-04-05 |

**Table S4:** Summary of S1 selected sequences from a replicate experiment with an independent RNA preparation of BVDV-7N/let-7. Some clones selected a 6mer sequence resulting in a 1-nt deletion; “_”.

| **Clone #** | **Total** | **S1 selection** |
| --- | --- | --- |
| 1, 11, 18, 19 | 4 | AUUUUGU |
| 2, 5, 7, 10 | 4 | _GAUGUU |
| 16,17 | 2 | CUGAGUG |
| 12, 15 | 2 | AGGUGUA |
| 4 | 1 | AUGUGGA |
| 6 | 1 | UUGUUGU |
| 8 | 1 | GUUUUUU |
| 9 | 1 | GUAAGGA |
| 3 | 1 | GUCUAGU |
| 13 | 1 | UAGUAGU |
| 14 | 1 | UUGAUGU |

**Table S5.** Summary of mutations acquired by BVDV wild-type and mutants after three passages to naïve cells. The location of mutations on the BVDV genome according to GenBank AJ133738 and corresponding amino acid changes are listed. Further differences exist between the NADL wild-type clone used and AJ133738. These are listed in Table S6 of reference (1); none of the clones used here contained premature stop-codons. Dashes indicate no changes and “+” indicates insertion of nucleotides to the viral genome. n.c.: non-coding.

| **BVDV variant** | **Position (nt)** | **RNA** | **Amino acid** | **Genomic region** |
| --- | --- | --- | --- | --- |
| wild-type | 2959, 9684 | A→U, C→A | Q→H, T→K | E2, NS5A |
| 2xlet-7 | 7707 | A→G | Y→C | NS5B |
| swap | 12458 | A→G | **-** | 3’ UTR |
| let-7/miR-21 | 12459 | A→G | **-** | 3’ UTR |
| 2xmiR-17 | 5957 | G→A | V→I | NS3 |
| UUUGUAG/let-7 | **-** | **-** | **-** | **-** |
| UUUGUAG/miR-17 | 6676 | A→G | n.c. | NS3 |
| let-7p3,4/miR-17 | 2959 | A→U | Q→H | E2 |
| let-7p3,4/let-7 | 2959, 3234 3238, 3242 3949, 7788 8051, 8768 9271, 9484 9992, 10710 | A→U, A→G A→G, A→G G→A, C→U A→G, A→C A→G, A→G A→G, G→A | Q→H, K→R I→M, K→E n.c., A→V T→A, K→Q  n.c., n.c.  I→V, n.c. | E2, E2 E2, E2  NS2, NS4B  NS4B, NS5A NS5A, NS5A NS5A, NS5B |
| let-7/let-7p3,4 | 12574 | +AG | **-** | 3’ UTR |

**SUPPLEMENTARY FIGURES**

**B**

**Fig S1. Auxiliary pairing importance for BVDV replication**

(**A**) Viral replication of BVDV-wild-type and BVDV- S2p3,4 after electroporation of MDBK cells measured by RT-qPCR on intracellular RNA normalized to RPS11 and the 4 hr timepoint. Trans-complemented miRNA mimics at 10nM is indicated. (**B**) Schematics of BVDV-wild-type, BVDV-2xlet-7 and BVDV-swap depicting miRNA binding to S1 and S2 and predicted auxiliary pairing. Numbering represents the positions on the miRNA were mutations were introduced for various experiments. miR-17 seed in dark green, miR-17 auxiliary pairing positions in light green, let-7i seed in dark purple, let-7i auxiliary pairing positions in light purple.

**Fig S2. BVDV mutants and their corresponding seed sites**

Summary of BVDV mutants identified and constructed for the study. In the first column, the name of the BVDV mutant is given, the genome representation is shown in the second column and the seed site is given in the third column. In the right panel, color coding for the genome schematics is depicted. The “AG” is an acquired di-nucleotide insertion four nts from the 3’ genomic terminus.

**Fig S3. BVDV bi-directional reporter map**

Schematics of the bi-directional reporter. The BVDV IRES was used to drive firefly luciferase expression (Fluc) and the NPHV IRES for renilla luciferase (Rluc). The 5’ UTR borders are indicated with black lines followed by the respective luciferase genes drawn in yellow and orange. The red line represents the SbfI restriction site used for digestion to produce the 3’ ends of the respective 3’ UTRs. The 3’ UTRs are indicated with a black line on their 5’ end. Cleavage at the SbfI site generates the exact BVDV 3’ end, whereas it leads to an additional 10 nts tail on the NPHV 3’ end (not shown).

**B**

| **BVDV** | **with S2 binding** | **BVDV** | **wo S2 binding** |
| --- | --- | --- | --- |
| wild-type | 40.8 | wild-type alt 1 | 30.8 |
|  |  | wild-type alt 2 | 27.8 |
| let-7p3,4/miR-17 | 40.8 | let-7p3,4/miR-17 alt 1 | 30.8 |
|  |  | let-7p3,4/miR-17 alt 2 | 27.8 |
| let-7/miR-17p3,4 | - | let-7/miR-17p3,4 alt 1 | 30.8 |
|  |  | let-7/miR-17p3,4 alt 2 | 27.8 |
| 2xlet-7 | 45.9 | 2xlet-7 | 29.4 |
| let-7p3,4/let-7 | 45.9 | let-7p3,4/let-7 | 29.4 |
| let-7/let-7p3,4 | - | let-7/let-7p3,4 | 29.4 |
|  |  | let-7/let-7p3,4 alt 1 | 27.1 |
| swap | 42.6 | swap | 26.1 |
|  |  | swap alt 1 | 25.5 |
| let-7/miR-21 | 35.12 | let-7/miR-21 | 29.4 |
|  |  | let-7/miR-21 alt 1 | 28 |
| 2xmiR-17 | 37.5 | 2xmiR-17 alt 1 | 27.5 |
|  |  | 2xmiR-17 alt 2 | 24.5 |
| let-7/miR-30 | 36.2 | let-7/miR-30 | 29.4 |
|  |  | let-7/miR-30 alt 1 | 27.8 |
| UUUGUAG/let-7 | 47.1 | UUUGUAG/let-7 alt 1 | 30.6 |
|  |  | UUUGUAG/let-7 alt 2 | 30 |
|  |  | UUUGUAG/let-7 alt 3 | 29.9 |
| UUUGUAG/miR-17 | 42 | UUUGUAG/miR-17 alt 1 | 32 |
|  |  | UUUGUAG/miR-17 alt 2 | 29 |
| poly-U-12500 | 40.8 | poly-U-12500 alt 1 | 30.8 |
|  |  | polyU-12500 alt 2 | 27.8 |
| poly-U-12518 | 40.6 | poly-U-12518 alt 1 | 30.6 |
| wt+AG | 37.7 | wt+AG alt 1 | 27.8 |
| ind | - | ind | 26.3 |
|  |  | ind alt 1 | 22.6 |
| miR-17/miR-17p3,4-UAAUUUCUC | - | miR-17/miR-17p3,4-UAAUUUCUC | 24.5 |

**Fig S4. Predicted structures of the 3’ UTR of BVDV wild-type and mutants**

(**A**) Schematics of BVDV-wild-type and mutants (nucleotides 12458-12578 of NADL genome) with or without miRNA binding at S2. Binding of S2 by the corresponding miRNA leads this region to remain single stranded. Since many structures resemble each other, common mutant structures were shown together. (**B**) Summary of the free folding energies for each predicted structure is given in -ΔG (kcal/kmol). Alternative structures are indicated with “alt” and a number for each alternative i.e. “alt 1”, “alt 2” etc. Folding energies of BVDV RNA and miRNAs together, were estimated by a simple addition of BVDV RNA folding energy (RNAeval) and intermolecular binding energy (IntaRNA). Structures were predicted on MFold and structure drawings were made with Varna (2-5).

**SUPPLEMENTARY REFERENCES**

1. Scheel, T.K., Luna, J.M., Liniger, M., Nishiuchi, E., Rozen-Gagnon, K., Shlomai, A., Auray, G., Gerber, M., Fak, J., Keller, I. *et al.* (2016) A Broad RNA Virus Survey Reveals Both miRNA Dependence and Functional Sequestration. *Cell Host Microbe*, **19**, 409-423.

2. Zuker, M. (2003) Mfold web server for nucleic acid folding and hybridization prediction. *Nucleic Acids Res*, **31**, 3406-3415.

3. Darty, K., Denise, A. and Ponty, Y. (2009) VARNA: Interactive drawing and editing of the RNA secondary structure. *Bioinformatics*, **25**, 1974-1975.

4. Hofacker, I.L. (2003) Vienna RNA secondary structure server. *Nucleic Acids Res*, **31**, 3429-3431.

5. Wright, P.R., Georg, J., Mann, M., Sorescu, D.A., Richter, A.S., Lott, S., Kleinkauf, R., Hess, W.R. and Backofen, R. (2014) CopraRNA and IntaRNA: predicting small RNA targets, networks and interaction domains. *Nucleic Acids Res*, **42**, W119-123.
